# Action of Nitrite of Amyl

**Published:** 1877-10

**Authors:** W. D. Lane


### ARTICLE VI.

### *Action of Nitrite of Amyl.*

Dr. W. D. Lane, in the *British Medical Journal*, Jan. 27, offers the following conclusions regarding the action of amyl nitrite:

1. Amyl nitrite when inhaled in small quantities, produces reddening of the face in man, and of the nose and mouth in kittens; this action is due, according to Brunton, to the dilatations and over-filling of the arterioles.

2. When inhaled by kittens in large quantities, it produces cyanosis of the nose and mouth along with insensibility. The cyanosis arises from over-distention of the venous system, this being due to the engorged arterioles propelling the blood into the veins, while the insensibility is probably caused by over-distention of the venous system and the heart.

3. When inhaled in small quantities, it produces recovery from chloroformic insensibility, by dilating the arterioles of the brain and thus removing the cerebral anemia due to chloroform.

4. When inhaled in large quantities, instead of producing recovery from chloroformic insensibility, it not only retards it, but it may cause death by paralysis and over distention of the heart, and engorgement of the venous system.

5. It causes a rise of temperature when inhaled in large quantities by the increased amount of blood in the arterioles, causing an increased tissue-change in the body.

9. In large doses (inhaled) it produces a fall of temperature.

7. It also helps to produce recovery from the chloroformic insensibility by raising the temperature which is always lowered by chloroform, and by removing the paralysis of the heart due to chloroform; this action is well seen by the nitrite of amyl making the heart's beats fewer and its sounds louder.

8. Death is caused chiefly by paralysis of the heart, which is shown by all its cavities being distended, and by engorgement of the venous system.—*Jour. Nervous and Mental Diseases.*

